# Validation of Potential Reference Genes for Real-Time qPCR Analysis in Pharaoh Ant, *Monomorium pharaonis* (Hymenoptera: Formicidae)

**DOI:** 10.3389/fphys.2022.852357

**Published:** 2022-02-28

**Authors:** Guo Ding, Qionghua Gao, Jun Chen, Jie Zhao, Guojie Zhang, Weiwei Liu

**Affiliations:** ^1^Section for Ecology and Evolution, Department of Biology, University of Copenhagen, Copenhagen, Denmark; ^2^State Key Laboratory of Genetic Resources and Evolution, Kunming Institute of Zoology, Chinese Academy of Sciences, Kunming, China; ^3^BGI-Shenzhen, Shenzhen, China; ^4^Guangxi Key Laboratory of Agric-Environment and Agric-Products Safety, College of Agriculture, Guangxi University, Nanning, China; ^5^School of Life Sciences, Yunnan University, Kunming, China

**Keywords:** *Monomorium pharaonis*, real-time quantitative PCR, RNA-seq, reference gene, expression stability, optimal reference gene number

## Abstract

Ants are highly diverse social insects living in colonies consisted of up to millions of individuals with reproductive division of labors. Due to the interests in disclosing the genetic and epigenetic regulation mechanisms underlying the distinct developmental trajectories between castes and division of labor in colonies, many ant species have recently been established as laboratory models for evolutionary development and social behavior studies. These functional studies often request a precise quantification of the relative gene expression level, which relies on a stably expressed reference genes for normalization. A core set of reliable reference genes for this purpose however has not been established yet in ants. In the present study, we tested the expression patterns and amplification efficiencies of 12 abundantly expressed candidate genes in *Monomorium pharaonis*, one of the few ant species that are suitable for laboratory rearing and experimentation. We quantified the expression levels of these genes by RT-qPCR in seven different conditions: embryo development, sexual development, worker development, adult phenotypes, tissues, and two abiotic manipulative treatments in pharaoh ant. Finally, five genes, elongation factor-1 alpha (*EF1A*), glyceraldehyde 3-phosphate dehydrogenase (*GAPDH*), TATA-box-binding protein (*TATA*), tubulin gamma-2 chain-like (*TBLg2*), heat shock protein 67B2-like (*HSP67*) were found to be the most stable reference genes across seven conditions. We also identified the most stable reference genes applicable for each distinct condition and the optimal number of reference genes entailed were evaluated. Our study validates reliable reference genes for RT-qPCR analysis which lays the foundation for future studies in pharaoh ant.

## Introduction

Social insects live in complex societies with sophisticated social behavior and division of labor. How natural selection shape their caste system has long fascinated biologists (Hölldobler and Wilson, 1990; Trible and Kronauer, 2017). The differentiated castes vary in morphology, physiology, and behavior even though they have the same genome (Abouheif and Wray, 2002; Li et al., 2014). With the morphologically and behaviorally distinct individuals, ant colony organizes into a complicated society with intricate division of labor among different castes (Hölldobler and Wilson, 1990). Many studies suggested that the caste differentiation is regulated by differentially expressed genes among castes (Evans and Wheeler, 2002; Pereboom et al., 2005; Khila and Abouheif, 2010; Qiu et al., 2018). Thus, interrogating the molecular mechanism underlying caste differentiation through the gene expression analysis has been the major focus in recent studies in social insects (Gospocic et al., 2017; Chandra et al., 2018).

Pharaoh ant (*Monomorium pharaonis*) is of high fecundity, short lifespan and polygynous (multiple queens in the same colony) colony organization, making it easier for lab rearing and suitable as a model organism to study the molecular mechanism of caste differentiation (Peacock and Baxter, 1949; Schmidt et al., 2011). The reproductive and non-reproductive castes can be distinguished via morphological traits since the 2nd instar larval stage: the worker larvae are furnished with numerous, bifurcated hairs and sexual (female reproductive and male) larvae are almost hairless; the sexual larvae are almost twice the size of worker larvae (Edwards, 1991). In addition, many differentially expressed genes among castes or individuals with different social roles have been identified by high-throughput sequencing and bioinformatics analysis in pharaoh ant (Mikheyev and Linksvayer, 2015; Qiu et al., 2018; Warner et al., 2019). However, studies on validation of the functions of differentially expressed genes in regulating caste phenotypes are lagging behind. The causal relationship linking gene expression with particular caste traits remains poorly understood.

Real-time quantitative polymerase chain reaction (RT-qPCR) is a popular method for analyzing gene expression level (Nolan et al., 2006; Derveaux et al., 2010). This technique can evaluate the expression level of target genes effectively by comparing to stable reference genes or by depicting standard curve of target genes (Bustin, 2000), with a lower cost and less time-consumption than RNA-seq. Therefore, it is a method still widely used to quantify gene expression even in the post-genomic era when transcriptome sequencing is readily available due to its high sensitivity and accuracy (Kang et al., 2017). However, the pipetting errors and differences in amplification efficiency among different samples can cause error for the threshold cycle (Ct, also called Cq), which is an important value to calculate the expression level of target genes. To minimize the error, stably expressed reference genes are necessary for gene expression analysis.

Reference genes are often used to normalize the expression level of target genes in different samples in RT-qPCR (Radonić et al., 2004). These genes are usually chosen from housekeeping genes, which are typically constitutive genes required for maintaining basic cellular functions, and are expressed in all cells at relatively constant levels (Butte et al., 2001; Zhu et al., 2008). Therefore, reference genes could be used to normalize the expression level of target genes among different samples to reduce or even eliminate systematic errors. Nevertheless, no universal gene can be applied to all situations as reference even in the same species (Cheng et al., 2013; Gao et al., 2017; Kang et al., 2017; Moreira et al., 2017; do Livramento et al., 2018). Given reference gene can directly affect gene expression profile analysis (do Livramento et al., 2018), making a thorough reference gene selection and validation prior to RT-qPCR analysis of target genes is necessary.

To date, the reference genes were only described in fire ant *Solenopsis invicta*, leaf-cutting ant *Atta sexdens rubropilosa* in detail among ant species (Cheng et al., 2013). In this study, we selected 12 genes as potential reference genes: 11 from our RNA-seq data and one commonly used reference gene outside of our data—18S ribosomal RNA (*18S*). The gene fragments were cloned into vector, and the amplification efficiency was calculated by standard curve method. Then we examined the expression stability of these genes in different developmental stages and tissues: (1) embryo development, (2) worker development, (3) sexual development, (4) adult phenotypes, (5) gyne/queen tissues (brain, midgut, fat body, and ovary); and two abiotic manipulative treatments for gyne/queen: (6) injection of eGFP dsRNA and (7) starvation treatment. In addition, the RT-qPCR results were evaluated with four methods [geNorm (Vandesompele et al., 2002), NormFinder (Andersen et al., 2004), BestKeeper (Pfaffl et al., 2004), and the comparative ΔCt method (Silver et al., 2006)]. Finally, the performance of these potential reference genes were integrated by a web-based comprehensive tool RefFinder (Xie et al., 2012). We validated the best reference genes and optimal gene numbers applicable to different developmental stages, adult phenotypes, tissues, and abiotic treatments for gene expression analysis in *M. pharaonis*, which provides a valuable guidance for future studies on functional validation of differentially expressed genes linking to caste phenotypes.

## Materials and Methods

### Biological Samples

The samples (eggs, larvae, pupae, and adults) were collected from colonies reared at Kunming Institute of Zoology and kept under a constant temperature of 27°C and 65% humidity. The samples used for abiotic treatments consisted of heads, thorax, and abdomens. All samples were collected in triplicate, flash froze in liquid nitrogen and stored at −80°C until use.

### Biotic Conditions

#### Embryo Development

2nd, 5th, and 8th day Eggs were collected. Since a single egg is too small for the RNA extraction, a pool of about 50 eggs was set as one sample to ensure high quality of RNA.

#### Worker Development

To obtain a stable reference gene during worker developmental stages, we collected 3rd instar worker larvae, young worker pupae, old worker pupae, and worker adult according to morphological features depicted by Berbdt and Kremer (1986). Given the tiny size of worker, 10 individuals were used for each worker sample.

#### Sexual Development

We collected 3rd instar sexual larvae. As the sex can be distinguished since the pupae stage, we prepared male/gyne young pupae, male/gyne old pupae, and male/gyne/queen adults, respectively. Gyne specifically refers to the virgin queen whose gene expression pattern is different from the reproductive active queen. Two individuals were enough for sexual samples because of their big size.

#### Adult Phenotypes

Here, we classified adult pharaoh ants based on their caste, reproductive condition and sex (Smith and Suarez, 2010). Four adult phenotypes were analyzed: gyne (unmated reproductive female), queen (mated, reproductively active female), worker (un-reproductive female) and male.

#### Tissues

Insemination induces drastic changes in physiology and behavior when a gyne becomes to a queen. Thus, tissue comparisons for reference genes between gyne and queen were also conducted. Individuals were dissected under a stereo microscope to obtain brain, midgut, fat body, and ovary. Each sample is consisted of a pool of twenty heads, thoraxes, and abdomens from gynes or queens, respectively.

### Abiotic Conditions

#### Injection

RNA interference (RNAi) is a popular method to interrogate gene functions. RNAi components are frequently administered to insects through micro injection technique. Micro injection would cause tissue damage to insect thus can be considered as an abiotic stress condition. We tested reference genes performance under micro injection treatment in which 1,000 ng/μL control eGFP dsRNA was injected into adult bodies. After 48 h, heads, thoraxes, and abdomens of injected and non-injected groups were, respectively, collected and analyzed.

#### Starvation

Starvation treatment is an abiotic stress condition which would elicit different behavioral responses in gyne and queen. After 5 days starvation treatment, heads, thoraxes, and abdomens of starved and control groups were, respectively, collected and analyzed.

### RNA Extraction and cDNA Synthesis

Total RNA was extracted using TRIzol Reagent (Invitrogen, United States) following the manufacturer’s instruction. RNA was eluted in 10–20 μL RNase-free water depending on the size of precipitate. The quality and concentration of RNA were measured using NanoDrop (Thermo Fisher Scientific, United States). Then 500 ng RNA was used to synthesize the first strand cDNA with Primescript RT reagent kit with gDNA Eraser (Perfect Real Time) (TaKaRa, Japan). Finally, all the cDNA samples were diluted 10-folds to be used as templates of RT-qPCR.

### Reference Gene Selection and Primer Design

The top 10 frequently used reference genes in insects were *Actin*, *RPL*, *Tubulin*, *GAPDH*, *RPS*, *18S*, *EF1A*, *TATA*, *HSP*, and *SDHA* (Lü et al., 2018). We picked out the genes, annotated as the homologs of these 10 widely used reference genes, from our RNA-seq data of embryonic stages (unpublished data). The low expression genes (TPM < 5 in at least 2 stages) were ruled out. Then, variance stabilizing transformation (VST) in R software (Anders and Huber, 2010) was used to eliminate the bias caused by expression level. Finally, the standard deviation of each gene was calculated. Except genes from our data, *18S* (18S ribosomal protein sequence, accession: JQ695802.1) and *NADH* (NADH-ubiquinone oxidoreductase subunit 8, accession: XM_012688246) were also added as potential candidate reference gene. Primers for each candidate gene were manually designed using Primer Premier 5.0 (Lalitha, 2002), and the quality of primers were evaluated by Oligo 7.0 (Rychlik, 2007; Table 1).

**TABLE 1:** The primer sets and amplification efficiency of the potential reference genes used for RT-qPCR.

Gene	Gene symbol	Primer sequence^a^ (5′–3′)	Amplicon length (bp)	Tm (°C)	E (%)^b^	R^2c^
Actin-5c	*ACT5C*	F: GTTGCCCTGAGGCTCTCTTCC	204	55	98.3	0.983
		R: GTAGACGGGGCTAAGGCAGTG				
28S ribosomal protein S23	*RPS23*	F: CCATTGTGGGTGGCAGTGTAT	129	55	106.4	0.988
		R: CTCTATGGAACCTTGCTCGGA				
60S ribosomal protein L5	*RPL5*	F: CGTGGGTAACACAAATGTAAATGG	200	55	97.9	0.984
		R: TCACTGTCGTAGCCAGGGAAT				
Tubulin gamma-2 chain-like	*TBLg2*	F: TTGAAAGTGAAAATGCCGTGC	153	55	105.9	0.993
		R: TTCTGTCCATTCCTTCTGTTGC				
Tubulin beta-1 chain	*TBLb1*	F: TACACTGGTGAGGGTATGGACG	235	55	95	0.987
		R: GTGTGTTGCGTTATCACTCGG				
Glyceraldehyde-3-phosphate dehydrogenase	*GAPDH*	F: GGCGTCAACTTGGAGGCTTAT	161	55	104	0.985
		R: GGTTTTCTGAGTAGCCGTAATCG				
Elongation factor 1-alpha	*EF1A*	F: TTCATTTATTGCTCTCACATCTACG	160	55	94.8	0.987
		R: ACCGTTGCCCTTTCTACTCTAA				
TATA-box-binding protein	*TATA*	F: CGAGTTAGCGTGGACGAGCAT	116	55	100.6	0.973
		R: ATAGGTTGTTTCAATCGGCGG				
Heat shock protein 67B2-like	*HSP67*	F: GAATACACCTTATCGGGAAACTGA	98	55	102	0.989
		R: GCATCCTTTTGTGCTTCTCGT				
Heat shock protein 83	*HSP83*	F: TAATCCTGCGAGAAAATGCCC	205	55	92.9	0.991
		R: GATGGGTCCGTAAGAGATTCATAT				
18S ribosomal RNA	*18S*	F: TGTCTCAGTGCATGCCGAAT	155	55	92.3	0.985
		R: AAGCGTCCCTTCCATCACTG				
NADH-ubiquinone oxidoreductase subunit 8	*NADH*	F: AGGTATTTCGAGAACCAGCG	199	55	10 0.9	0.992
		R: GTCCTTCGAGATCCATCTGC				

*^a^F and R indicate forward and reverse primers, respectively.*

*^b^RT-qPCR efficiency, calculated by the standard curve method.*

*^c^Determination coefficient.*

### Real-Time Quantitative PCR

RT-qPCR was conducted using CFX96 Touch Real-time PCR Detection System (Bio-Rad, United States) based on TB Green Premix Ex Taq (Tli RNaseH Plus) (TaKaRa, Japan). Amplifications were carried out under the following conditions: initial denaturation at 95°C for 5 min followed by 40 cycles of 20 s at 95°C, for 20 s at 55°C, and for 20 s at 72°C. This was followed by 1 min at 95°C and 1 min at 50°C. Finally, a melting curve analysis with the temperature raised from 65 to 95°C in sequential steps of 0.5°C for 10 s. The reaction system is 20 μL, including 10 μL TB Green Premix EX Taq polymerase, 0.5 μL of each primer, 2 μL cDNA and 7 μL ddH_2_O. For each biological sample, three technical replicates were performed and the average cycle quantification (Cq) value were calculated. To create the standard curve, the amplicons were cloned into pMD-19T vector (TaKaRa, Japan). Then six 10-fold serial dilutions (from 1e + 4 to 1e + 9 copy number per microliter) were made according to copy number of the plasmids. With the dilutions as templates to conduct RT-qPCR, the standard curves were made. The amplification efficiency (E) values were calculated based on the equation: E = [10^(–1/slope)^-1] × 100 (Radonić et al., 2004), where slope is the slope of the standard curve.

### Data Analysis

The average Cq values were used for data analysis. Four algorithms including geNorm, NormFinder, BestKeeper and comparative ΔCt method, were used to evaluate the expression stability of the 12 potential reference genes. Finally, RefFinder was used to conduct a comprehensive evaluation and screen the optimal reference genes. The optimal number of reference genes for normalization was determined by geNorm.

## Results

### Reference Gene Selection

In our RNA-seq data, none of the genes was annotated as 18S and SDHA, two of the top 10 candidate genes in insects; 205 genes were annotated as the homologs of the other eight reference genes (Supplementary Table 1). After ruling out low expression genes according to the quantification with RNA-seq, 175 genes were left (Supplementary Table 2). Then we ranked the homologs of each gene according to their standard deviation across 16 embryonic stages (Supplementary Table 3). Finally, we chose potential reference genes with two criteria if one gene has more than one homolog: (1) have the low standard deviation among the homologs; (2) the TPM value should larger than 1,000 in at least eight stages. Addition to *18S*, annotated in NCBI, and *NADH*, validated in ants (do Livramento et al., 2018; Rajakumar et al., 2018), we obtained a list of 12 candidate reference genes for the subsequent experiments (Table 1).

### Transcriptional Profiling of Candidate Reference Genes

Firstly, the specificity of these reference genes was investigated by RT-PCR. All genes had a single band of the expected size on 1% agarose gel. Sequencing results showed that these amplicons were identical to corresponding gene sequences. Also, they had single peaks in melting-curve analysis in RT-qPCR (Supplementary Figure 1). The amplification efficiency for each gene ranged from 92.3 to 106.4%, showing a good amplification performance (Table 1). And all standard curves are shown in Supplementary Figure 2.

**FIGURE 1:**
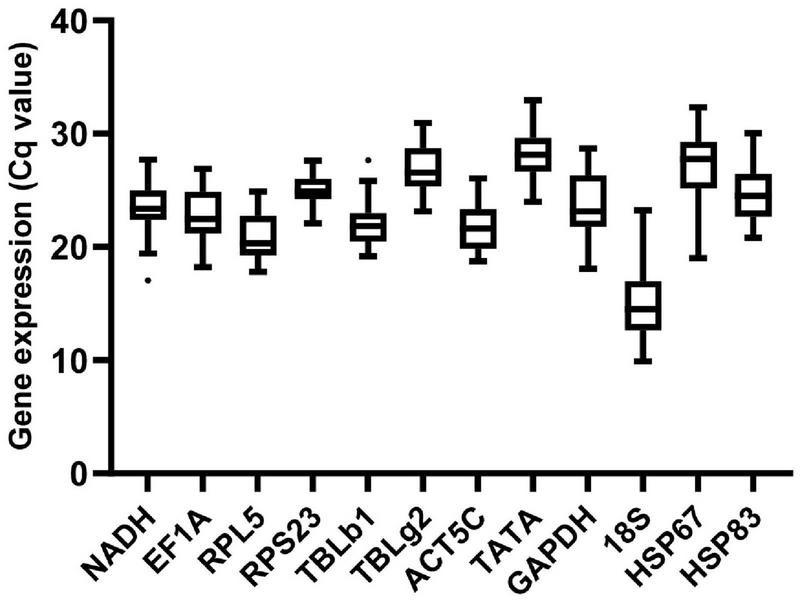
Expression profiles of reference genes in all 36 samples in *M. pharaonis.* The expression level of reference genes is presented with Cq value. The dots indicate outliers.

Among all samples for seven conditions, gene expression analysis of the 12 candidate reference genes presented a broad range of variance of Cq values from 9.57 (*18S*) to 33.03 (*TATA*) (Figure 1). Of the 12 reference genes, *18S* was the most abundant transcripts, and *TATA* was expressed at the lowest level.

### Expression Stability of Potential Reference Genes

#### Reference Gene Expression Stability During Embryonic Development

**FIGURE 2:**
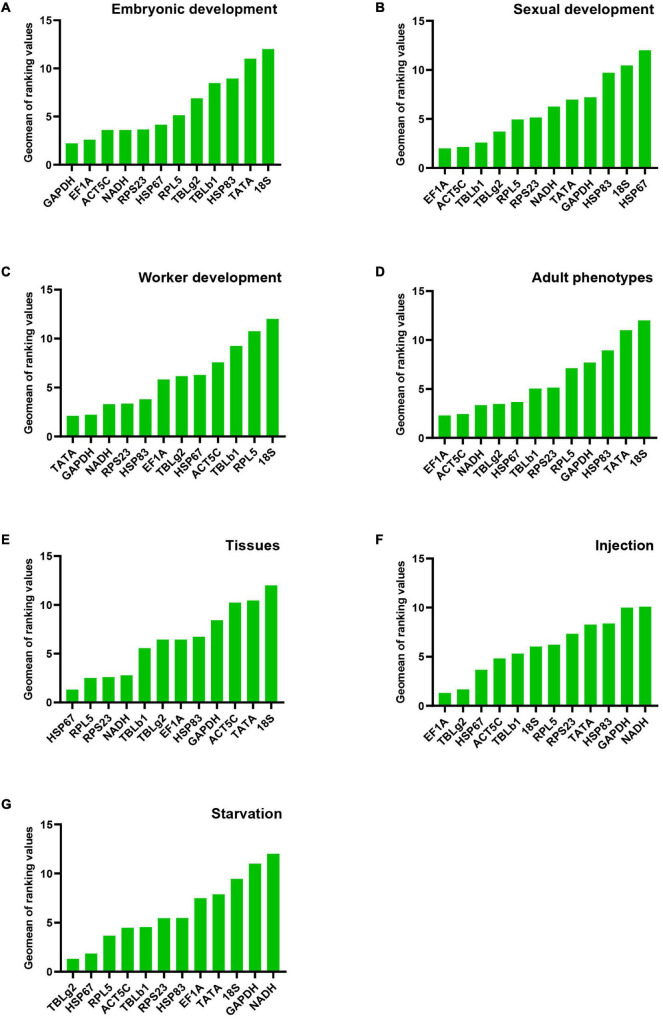
Stability of candidate reference gene expression in *M. pharaonis* in conditions of embryonic development **(A)**, sexual development **(B)**, worker development **(C)**, adult phenotypes **(D)**, tissues **(E)**, injection **(F)**, and starvation **(G)**. The stability of reference gene expression was calculated by RefFinder, which summarizing four different algorithms (geNorm, Norm finder, BestKeeper, and comparitive ΔCt). A lower value means more stable expression.

All analysis programs ranked *TATA* and *18S* as the least stable genes. However, the programs indicated different candidates for the most stable gene. *GAPDH* was the most stable gene in comparative ΔCt and NormFinder. However, geNorm indicated both *NADH* and *EF1A* are the most stable gene. The most stable gene *RPL5*, calculated by BestKeeper, only ranked in the 10th position in comparative ΔCt and NormFinder (Table 2). Integration of the results by RefFinder gives an overall stability ranking: *GAPDH* > *EF1A* > *ACT5C* > *NADH* > *RPS23* > *HSP67* > *RPL5* > *TBLg2* > *TBLb1* > *HSP83* > *TATA* > *18S* (Figure 2A). This order is different from that according to RNA-seq data: *RPS23* > *HSP67* > *NADH* > *RPL5* > *EF1A* > *TATA* > *TBLg2* > *HSP83* > *TBLb1* > *GAPDH* > *ACT5C* (Supplementary Table 3).

**TABLE 2:** Stability of reference gene expression in different stages and tissues.

Biotic	Gene	geNorm	Norm Finder	Best Keeper	Comparative ΔCt
Condition		Stability (Rank)	Stability (Rank)	Stability (Rank)	Stability (Ranking)
Embryonic development	*GAPDH*	0.218 (4)	0.043 (1)	0.421 (6)	0.73 (1)
	*EF1A*	0.129 (1)	0.433 (5)	0.294 (3)	0.774 (3)
	*ACT5C*	0.309 (6)	0.167 (2)	0.481 (7)	0.773 (2)
	*NADH*	0.129 (1)	0.51 (7)	0.326 (4)	0.827 (6)
	*RPS23*	0.177 (3)	0.503 (6)	0.206 (2)	0.797 (5)
	*HSP67*	0.275 (5)	0.352 (3)	0.378 (5)	0.78 (4)
	*RPL5*	0.41 (7)	1.074 (10)	0.144 (1)	1.18 (10)
	*TBLg2*	0.604 (9)	0.365 (4)	0.896 (9)	0.938 (7)
	*TBLb1*	0.513 (8)	0.844 (9)	0.677 (8)	1.127 (9)
	*HSP83*	0.695 (10)	0.727 (8)	1.051 (10)	1.079 (8)
	*TATA*	0.811 (11)	1.158 (11)	1.357 (11)	1.376 (11)
	*18S*	1.055 (12)	2.205 (12)	1.982 (12)	2.273 (12)
Sexual development	*EF1A*	0.531 (1)	0.682 (4)	1.402 (2)	1.524 (2)
	*ACT5C*	0.678 (3)	0.181 (1)	1.803 (7)	1.469 (1)
	*TBLb1*	0.531 (1)	0.626 (3)	1.573 (5)	1.554 (3)
	*TBLg2*	0.788 (4)	0.465 (2)	1.742 (6)	1.565 (4)
	*RPL5*	0.876 (5)	1.245 (8)	1.507 (3)	1.812 (5)
	*RPS23*	1.136 (7)	1.656 (10)	0.764 (1)	2.127 (10)
	*NADH*	1.26 (8)	1.168 (6)	1.518 (4)	1.902 (8)
	*TATA*	1.001 (6)	1.221 (7)	1.936 (8)	1.885 (7)
	*GAPDH*	1.365 (9)	1.083 (5)	2.476 (10)	1.859 (6)
	*HSP83*	1.451 (10)	1.318 (9)	2.723 (11)	1.994 (9)
	*18S*	1.757 (11)	3.048 (11)	2.464 (9)	3.3 (11)
	*HSP67*	2.035 (12)	3.206 (12)	3.661 (12)	3.424 (12)
Worker development	*TATA*	0.614 (5)	0.244 (2)	0.601 (2)	1.01 (1)
	*GAPDH*	0.484 (3)	0.235 (1)	0.753 (4)	1.072 (2)
	*NADH*	0.423 (1)	0.642 (5)	1.02 (8)	1.111 (3)
	*RPS23*	0.693 (7)	0.245 (3)	0.379 (1)	1.164 (6)
	*HSP83*	0.423 (1)	0.704 (7)	0.994 (6)	1.163 (5)
	*EF1A*	0.744 (8)	0.631 (4)	1.032 (9)	1.15 (4)
	*TBLg2*	0.656 (6)	0.604 (6)	0.892 (5)	1.253 (8)
	*HSP67*	0.568 (4)	0.793 (8)	1.006 (7)	1.18 (7)
	*ACT5C*	0.982 (11)	0.96 (10)	0.707 (3)	1.527 (10)
	*TBLb1*	0.979 (9)	0.941 (9)	1.252 (10)	1.305 (9)
	*RPL5*	0.894 (10)	1.329 (11)	1.315 (11)	1.597 (11)
	*18S*	1.437 (12)	3.657 (12)	1.883 (12)	3.71 (12)
Adult phenotypes	*EF1A*	0.415 (4)	0.132 (1)	1.163 (7)	0.824 (1)
	*ACT5C*	0.123 (1)	0.354 (3)	1.086 (6)	0.873 (2)
	*NADH*	0.123 (1)	0.508 (5)	1.071 (5)	0.932 (5)
	*TBLg2*	0.272 (3)	0.515 (6)	0.837 (2)	0.919 (4)
	*HSP67*	0.462 (5)	0.38 (4)	0.952 (3)	0.888 (3)
	*TBLb1*	0.559 (6)	0.351 (2)	1.516 (9)	0.939 (6)
	*RPS23*	0.664 (7)	1.115 (10)	0.502 (1)	1.282 (10)
	*RPL5*	0.723 (8)	1.045 (9)	0.957 (4)	1.25 (9)
	*GAPDH*	0.805 (9)	0.898 (7)	1.441 (8)	1.197 (7)
	*HSP83*	0.894 (10)	0.9 (8)	1.920 (10)	1.215 (8)
	*TATA*	1.008 (11)	1.402 (11)	1.929 (11)	1.572 (11)
	*18S*	1.142 (12)	1.692 (12)	2.381 (12)	1.815 (12)
Tissues	*HSP67*	0.568 (3)	0.418 (1)	0.768 (1)	1.012 (1)
	*RPL5*	0.506 (1)	0.718 (5)	0.823 (2)	1.133 (4)
	*RPS23*	0.506 (1)	0.624 (3)	0.956 (5)	1.084 (3)
	*NADH*	0.687 (5)	0.460 (2)	0.827 (3)	1.074 (2)
	*TBLb1*	0.627 (4)	0.731 (6)	1.063 (8)	1.145 (5)
	*TBLg2*	0.918 (8)	0.708 (4)	1.069 (9)	1.218 (6)
	*EF1A*	0.759 (6)	0.980 (9)	0.856 (4)	1.307 (8)
	*HSP83*	0.828 (7)	0.809 (7)	0.957 (6)	1.234 (7)
	*GAPDH*	1.072 (10)	0.975 (8)	1.013 (7)	1.385 (9)
	*ACT5C*	1.132 (11)	1.164 (10)	1.326 (10)	1.487 (10)
	*TATA*	0.996 (9)	1.213 (11)	1.559 (11)	1.488 (11)
	*18S*	1.318 (12)	2.118 (12)	1.831 (12)	2.249 (12)

#### Reference Gene Expression Stability During Sexual Development

For the stability ranking, four software showed distinct orders due to different algorithms. *ACT5C* was regarded the most stable reference gene in comparative ΔCt and NormFinder. However, it ranked 7th in BestKeeper. *HSP67* was the least stable reference gene during sexual development in four programs (Table 2). RefFinder was used to integrate the results of the four programs. The overall stability ranking of the reference genes is: *EF1A* > *ACT5C* > *TBLb1* > *TBLg2* > *RPL5* > *RPS23* > *NADH* > *TATA* > *GAPDH* > *HSP83* > *18S* > *HSP67* (Figure 2B).

#### Reference Gene Expression Stability During Worker Development

Data from four developmental stages of worker, 3rd instar larvae, young pupae, old pupae, and adults, were used to screen the stable reference genes. *NADH* and *HSP83*, *GAPDH*, *RPS23*, *TATA* were considered as the most stable reference genes by geNorm, NormFinder, BestKeeper, and comparative ΔCt, respectively (Table 2). The overall stability ranking of the reference genes by RefFinder is: *TATA* > *GAPDH* > *NADH* > *RPS23* > *HSP83* > *EF1A* > *TBLg2* > *HSP67* > *ACT5C* > *TBLb1* > *RPL5* > *18S* (Figure 2C).

#### Reference Gene Expression Stability Across Adult Phenotypes

All analysis programs indicated that *18S* was the least stable gene. However, they showed different choices for the most stable gene. *EF1A* was regarded as the most stable reference gene in comparative ΔCt and NormFinder. However, it was ranked at 7th position in BestKeeper (Table 2). RefFinder was used to integrate the results of the four programs. The overall stability ranking of the reference genes by RefFinder is: *EF1A* > *ACT5C* > *NADH* > *TBLg2* > *HSP67* > *TBLb1* > *RPS23* > *RPL5* > *GAPDH* > *HSP83* > *TATA* > *18S* (Figure 2D).

#### Reference Gene Expression Stability Across Gyne/Queen Tissues

All analysis programs indicated *18S* is the least stable genes among the brain, midgut, fat body, and ovary of gyne/queen, but they showed different results for the most stable gene. Comparative ΔCt, NormFinder, and BestKeeper suggested *HSP67* is the most stable gene, while both *RPS23* and *RPL5* were presented as the most stable gene in geNorm (Table 2). According to RefFinder, the overall stability of the reference genes was ranked as follows: *HSP67* > *RPL5* > *RPS23* > *NADH* > *TBLb1* > *TBLg2* > *EF1A* > *HSP83* > *GAPDH* > *ACT5C* > *TATA* > *18S* (Figure 2E).

#### Reference Gene Expression Stability in Injection Treatment

Injection of eGFP dsRNA results in the change of stability ranking among these genes. All programs, except BestKeeper, suggested *EF1A* is the most stable gene. While BestKeeper indicated *18S* is the most stable gene (Table 3). According to RefFinder, the overall stability ranking of the reference genes is *EF1A* > *TBLg2* > *HSP67* > *ACT5C* > *TBLb1* > *18S* > *RPL5* > *RPS23* > *TATA* > *HSP83* > *GAPDH* > *NADH* (Figure 2F).

**TABLE 3:** Stability of reference gene expression under abiotic conditions.

Abiotic condition	Gene	geNorm	Norm Finder	Best Keeper	Comparative ΔCt
		Stability (Ranking)	Stability (Ranking)	Stability (Ranking)	Stability (Ranking)
Injection	*EF1A*	0.249 (1)	0.125 (1)	0.907 (3)	0.844 (1)
	*TBLg2*	0.249 (1)	0.177 (2)	0.767 (2)	0.887 (2)
	*HSP67*	0.38 (3)	0.347 (3)	0.944 (5)	0.934 (4)
	*ACT5C*	0.429 (4)	0.425 (5)	1.201 (9)	0.911 (3)
	*TBLb1*	0.457 (5)	0.353 (4)	1.136 (8)	0.941 (5)
	*18S*	1.053 (11)	1.492 (11)	0.588 (1)	1.721 (11)
	*RPL5*	0.521 (6)	0.674 (6)	1.047 (7)	1.013 (6)
	*RPS23*	0.719 (9)	0.968 (9)	0.908 (4)	1.242 (9)
	*TATA*	0.592 (7)	0.947 (8)	1.527 (12)	1.181 (7)
	*HSP83*	0.654 (8)	0.872 (7)	1.393 (11)	1.186 (8)
	*GAPDH*	0.909 (10)	1.352 (10)	1.346 (10)	1.589 (10)
	*NADH*	1.197 (12)	1.783 (12)	1.042 (6)	1.918 (12)
Starvation	*TBLg2*	0.264 (1)	0.129 (1)	1.676 (3)	1.144 (1)
	*HSP67*	0.264 (1)	0.485 (4)	1.495 (1)	1.219 (3)
	*RPL5*	0.647 (5)	0.171 (2)	2.023 (9)	1.183 (2)
	*ACT5C*	0.562 (4)	0.624 (5)	1.760 (4)	1.243 (4)
	*TBLb1*	0.486 (3)	0.768 (6)	1.760 (4)	1.307 (6)
	*RPS23*	0.914 (9)	0.924 (7)	1.502 (2)	1.479 (7)
	*HSP83*	0.696 (6)	0.306 (3)	2.037 (10)	1.249 (5)
	*EF1A*	0.790 (7)	1.250 (8)	1.926 (7)	1.556 (8)
	*TATA*	0.857 (8)	1.304 (9)	1.899 (6)	1.620 (9)
	*18S*	1.183 (10)	2.124 (10)	1.974 (8)	2.398 (10)
	*GAPDH*	1.438 (11)	2.209 (11)	2.436 (11)	2.402 (11)
	*NADH*	1.602 (12)	2.242 (12)	2.714 (12)	2.422 (12)

#### Reference Gene Expression Stability in Starvation Treatment

For starvation treatment, the stability ranking was also changed compared with non-starvation control group. All analysis programs, except BestKeeper, indicated *TBLg2* was the most stable gene (Table 3). According to RefFinder, the overall stability ranking of the reference genes is *TBLg2* > *HSP67* > *RPL5* > *ACT5C* > *TBLb1* > *RPS23* > *HSP83* > *EF1A* > *TATA* > *18S* > *GAPDH* > *NADH* (Figure 2).

#### Optimal Number of Reference Genes

Significant errors can be caused by using inappropriate number of reference genes (do Livramento et al., 2018). The minimal number of reference genes necessary for normalization is determined by *V*-value (pairwise variation) in geNorm program. *V*-value is calculated by two neighboring normalization factors (NF). Usually, a value below 0.15 is considered ideal but it depends on situation. However, 0.15 must not to be taken as a too strict cut-off. In some cases, the trend is also equally informative (Vandesompele et al., 2002).

**FIGURE 3:**
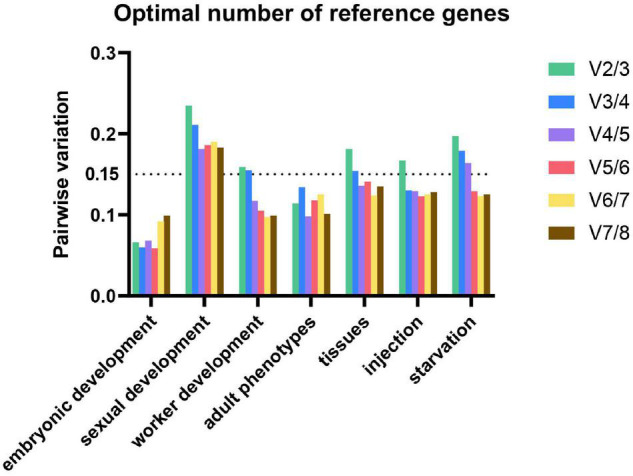
Optimal number of reference genes for normalization in *M. pharaonis.* The pairwise variation (V_n_/V_n+1_) was analyzed between the normalization factors NF_n_ and NF_n+1_ by geNorm. Average value of pairwise variations (v) indicates the optimal number of reference genes. The values that above the threshold value of 0.15 means an extra reference gene should add to the stability of the normalization factor.

In our study, the optimal numbers of reference genes are various under different experimental conditions. Using 0.15 as a threshold value, the optimal number of reference genes for worker development, tissues, injection, and starvation are 4, 4, 3, and 5, respectively. For gene expression analysis in embryonic development and adult phenotypes, the V values were all below 0.15, thus the observed trend (of changing *V*-values when using additional genes) can be used as guidance. Three and two reference genes are needed for embryonic development and adult phenotypes, respectively. As to sexual development, four reference genes are required for normalization. Therefore, we must make a balance between efficiency and accuracy (Figure 3).

## Discussion

RT-qPCR is a widely used method to evaluate the gene expression levels because of its low cost and high sensitivity. Especially in the genomic era, differentially expressed genes identified by RNA-seq analysis need to be verified by RT-qPCR (Bonasio et al., 2010; Gospocic et al., 2017). In our study, the stability ranking of reference genes was calculated according to RNA-seq data and RT-qPCR data. The different stability ranking indicate that RT-qPCR verification is indispensable even that RNA-seq data can provide a wealth of quantitative gene expression information. On the other hand, RT-qPCR data can improve our efficiency to choose potential candidate genes. It is not easy to make choice manually among 205 potential candidate genes. To minimize the variation and errors in RT-qPCR, data normalization is essential to obtain accurate gene expression data.

Some genes have already been frequently used as reference genes in RT-qPCR experiment (Lü et al., 2018). However, it is impossible to find a universal reference gene applicable to all species since the gene can participate in different cellular functions in different species (Moreira et al., 2017; do Livramento et al., 2018). *NADH* has been validated as a stable reference gene for RT-qPCR in several hymenopteran species (do Livramento et al., 2018; Jia et al., 2018; Rajakumar et al., 2018), but our data shows that it is not suitable for some situation of *M. pharaonis*. Especially, for gene expression analysis of neural plasticity between gyne and queen tissues, *NADH* is the least stable gene among the 12 reference genes. Since reference gene directly affects the results of gene expression analysis, it is necessary to do a comprehensive selection and validation of reference genes in *M. pharaonis* in different conditions.

Previous studies showed that only one reference gene was used in some gene expression analysis in ant, such as ribosomal protein L32 in *Camponotus floridanus* (Kupper et al., 2016) and β-actin in *Polyrhachis vicina* Roger (Shumin et al., 2010; Liu et al., 2013). However, the amplification efficiency of single reference gene was not mentioned in these studies. Our study gives the first insights into the knowledge of reference genes in *M. pharaonis*, which provides a valuable guideline for future studies interrogating molecular basis underlying division of labor in ants.

Notably, this is the first study to evaluate reference genes under abiotic treatments in ants. The treatment of injection and starvation are often used to study the genes or nutritional factors influencing queen reproductive ability. Thus, Whether the expression of reference genes is influenced by the abiotic treatment need to be validated. Although the stability rankings under different treatments are not the same as the control, the orders do not change dramatically. The most stable genes (*HSP67*, *RPL5*, and *ACT5C*) in control group, still show good performance in abiotic treatment groups.

In conclusion, we evaluated performance of 12 candidate reference genes in *M. pharaonis* under different situations by using four different methods. Consensus lists for gene stability were obtained and the optimal reference gene numbers were recommended under different situations. The result presented here is essential for gene expression analysis and will guide future gene function studies in pharaoh ant.

## Data Availability Statement

The data presented in the study are deposited in the NCBI GEO repository (https://www.ncbi.nlm.nih.gov/geo/), accession number BioProject PRJNA767561.

## Author Contributions

GD, GZ, and WL designed the study. JZ collected the samples. GD, QG, and JC performed the experiments and collected the data. GD analyzed the data. GD, QG, and WL wrote the manuscript. All authors read and approved the final version of the manuscript.

## Conflict of Interest

GD and GZ were employed by BGI-Shenzhen. The remaining authors declare that the research was conducted in the absence of any commercial or financial relationships that could be construed as a potential conflict of interest.

## Publisher’s Note

All claims expressed in this article are solely those of the authors and do not necessarily represent those of their affiliated organizations, or those of the publisher, the editors and the reviewers. Any product that may be evaluated in this article, or claim that may be made by its manufacturer, is not guaranteed or endorsed by the publisher.
