# Supplementary material for: Validation of Potential Reference Genes for Real-Time qPCR Analysis in Pharaoh Ant, Monomorium pharaonis (Hymenoptera: Formicidae)
Source: Front Physiol. 2022 Feb 28;13:852357. doi: 10.3389/fphys.2022.852357 (PMC8919206; doi:10.3389/fphys.2022.852357)
Supplement: Supplementary file 1 [file Data_Sheet_1.zip › data sheet 1/Supplementary figure 2_standard curves.pdf]

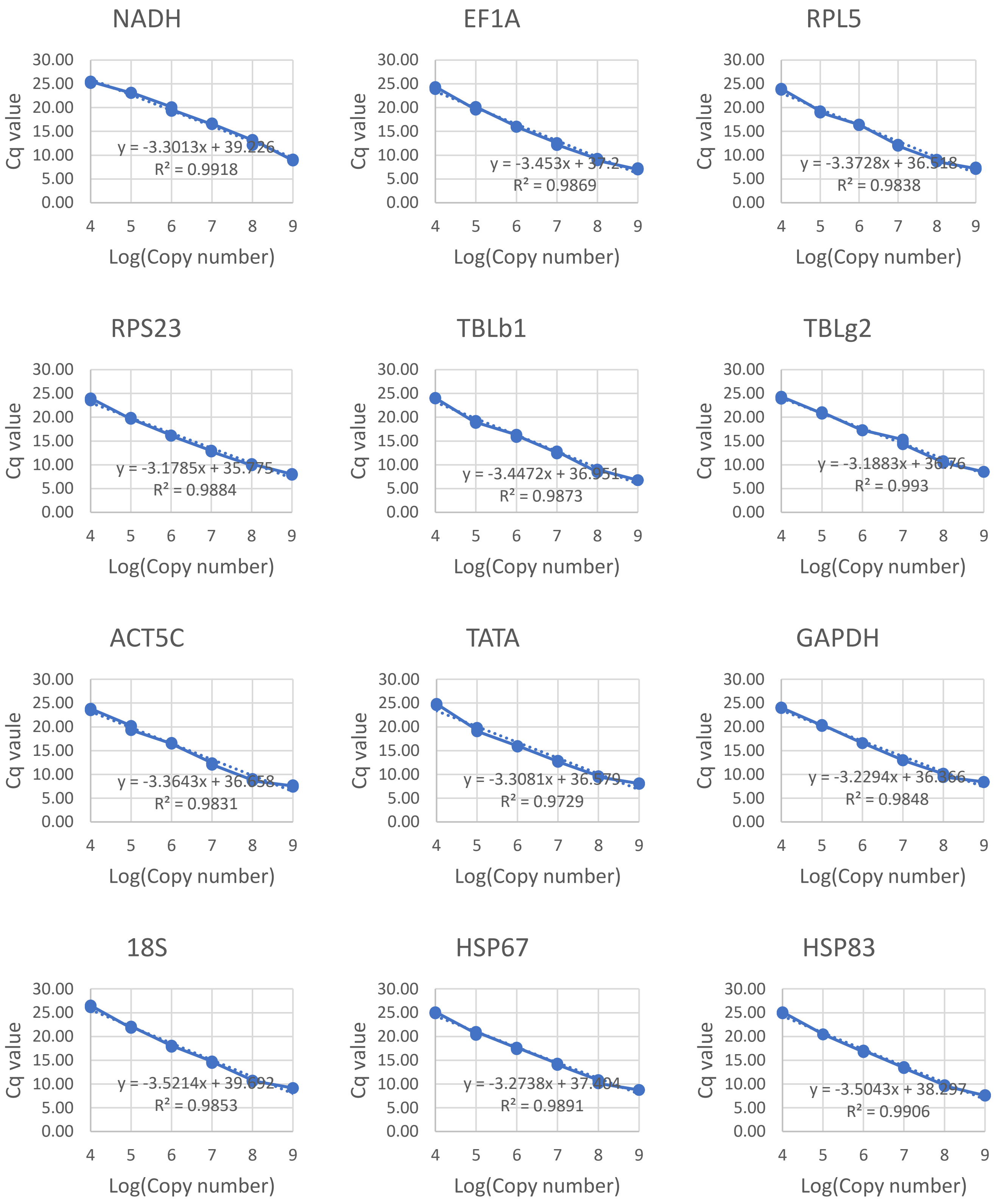

Supplementary Figure 2. standard curves of the 12 candidate reference genes in *M. pharaonis*.
